# Morphological and molecular characteristics of *Parasitodiplogaster religiosae* n. sp. (Nematoda: Diplogastrina) associated with *Ficus religiosa* in China

**DOI:** 10.1371/journal.pone.0199417

**Published:** 2018-07-11

**Authors:** Yongsan Zeng, Wensheng Zeng, Yuan Zhang, Weimin Ye, Dongmei Cheng, Natsumi Kanzaki, Robin M. Giblin-Davis

**Affiliations:** 1 Department of Plant Protection, Zhongkai University of Agriculture and Engineering, Guangzhou, People’s Republic of China; 2 Nematode Assay Section, Agronomic Division, North Carolina Department of Agriculture & Consumer Services, Raleigh, North Carolina, United States of America; 3 Kansai Research Center, Forestry and Forest Products Research Institute, Kyoto, Japan; 4 Fort Lauderdale Research and Education Center, Department of Entomology and Nematology, University of Florida-IFAS, Fort Lauderdale, Florida, United States of America; Aarhus University, DENMARK

## Abstract

A new nematode species of the genus *Parasitodiplogaster* was recovered from syconia of *Ficus religiosa* at the Guangxiao Temple, Guangzhou, China. It is described herein as *P*. *religiosae* n. sp. and is characterised by possessing the longest and thinnest spicule of all currently described males in the genus, an elongated laterally “ε-shaped” and ventrally rhomboid-like gubernaculum, a stoma without teeth, consisting of a ring-like cheilostom with indistinct anteriolateral projections, a tube-like gymnostom and a funnel-like stegostom, monodelphic with a mean vulval position of 66%. There are three pre-cloacal and six post-cloacal male genital papillae with the arrangement P1, P2, P3, (C, P4), P5, P6d, P7, P8, P9d, Ph. This new species was easily differentiated from other members of the genus by DNA sequences of partial small subunit rRNA gene (SSU) and the D2-D3 expansion segments of the large subunit rRNA gene (LSU). Phylogenetic analysis also corroborated its reasonable placement within a well-supported monophyletic clade with other *Parasitodiplogaster* species and within the *australis*-group that includes *P*. *australis* and *P*. *salicifoliae* that are all associates of fig wasp pollinators (*Platyscapa* sp.) of figs of the subsection Urostigma.

## Introduction

*Ficus religiosa* L., a monoecious fig, popularly known as the ashwattha tree, bo tree, bodhi tree, peepal tree, peepul tree, pippala tree or sacred fig, belongs to the genus *Ficus*, subgenus *Urostigma*, section *Urostigma*, and subsection *Urostigma*. It is native to India, southwest China and Mainland Southeast Asia, and has been introduced and cultivated worldwide as an ornamental tree in parks and gardens in subtropical and tropical areas with the potential for becoming an invasive weed, if its pollinator is co-introduced and becomes established (https://www.cabi.org/isc/datasheet/241680). It is also grown for traditional medicine for various types of human disorders, such as asthma, diabetes, diarrhea, epilepsy, gastric problems, inflammation, infections and sexual disorders[1]. It is pollinated by the fig wasp, *Platyscapa quadraticeps* (Mayr) (Agaonidae)(http://www.figweb.org/Ficus/Subgenus_Urostigma/Section_Urostigma/Subsection_Urostigma/Ficus_religiosa.htm).

*Parasitodiplogaster* Poinar, 1979 was described as the first parasitic nematode genus associated with fig-pollinating wasps by Poinar[2]. There is an interesting fact that the nematodes *Parasitodiplogaster* (Diplogasteridae) and *Schistonchus* (Aphelenchoididae) which are vectored by pollinator wasps are in apparently species-specific associations in co-evolved complex of nematode parasites and fig wasp pollinators[3]. The genus *Parasitodiplogaster* has since been reported from fig wasps and the sycones of *Ficus* species in North and Central America, Africa and Australia with a current total of 16 described species including *P*. *australis* Bartholomaeus, Davies, Ye, Kanzaki & Giblin-Davis, 2009, *P*. *citrinema* Poinar & Herre, 1991, *P*. *doliostoma* Kanzaki, Giblin-Davis, Davies & Center, 2012, *P*. *duganema* Poinar & Herre, 1991, *P*. *laevigata* Giblin-Davis, Ye, Kanzaki, Williams, Morris & Thomas, 2006, *P*. *maxinema* Poinar & Herre, 1991, *P*. *nymphanema* Poinar & Herre, 1991, *P*. *obtusinema* Poinar & Herre, 1991, *P*. *paranema* Poinar & Herre, 1991, *P*. *pertanema* Poinar & Herre, 1991, *P*. *pharmaconema* Kanzaki, Giblin-Davis, Ye, Herre & Center, 2013, *P*. *popenema* Poinar & Herre, 1991, *P*. *salicifoliae* Wöhr, Greeff, Kanzaki & Giblin-Davis, 2015, *P*. *sycophilon* Poinar, 1979, *P*. *trigonema* Poinar & Herre, 1991 and *P*. *yoponema* Poinar & Herre, 1991 [2,4–9]. Three morphospecies (*Msp*1-3) of *Parasitodiplogaster* associated with figs from the subgenus *Pharmacosycea* in Panama were discriminated by male tail characters and *Pharmacosycea*-associated *Parasitodiplogaster* species were referred to as the *P*. *maxinema-*group by genotype[10]. Kanzaki et al.[11] re-characterised three *Parasitodiplogaster* species (*P*. *nymphanema*, *P*. *obtusinema* and *P*. *trigonema*) from species of *Ficus* from the subgenus Urostigma, section Urostigma, and subsection Americana in Panama based on morphological and molecular profiles, and revealed that these three species belong to the *P*. *laevigata*-group because of molecular phylogenetic inferences and some typological characters, *e*.*g*., stomatal morphology. Until now, no *Parasitodiplogaster* species have been reported from figs from China. A recent survey on diversity of fig-associated nematodes in the Guangdong Province from 2016 to 2017 revealed an undescribed species of diplogastrid nematode from *F*. *religiosa* in Guangzhou, China. It is described here as *Parasitodiplogaster religiosae* n. sp. using morphological and molecular methods.

## Material and methods

### Ethics statement

Specific permissions were not required for the nematodes collected for the present study in Guangdong Province, China. The field used for nematode collection was not privately owned but open to the public and did not involve endangered or protected species.

### Nematode materials

Syconia in phase B-C were collected from a *F*. *religiosa* tree from the Guangxiao Temple in Guangzhou, China on May 26, 2016. They were dissected open with a scalpel and placed in distilled water for 20 min. Nematodes were then handpicked alive into water for DNA extraction, amplification, and sequencing attempts, or collected, heat-killed at 65°C for 2–3 min and placed into 4% formalin for measurements by light microscopy and then processed into 100% glycerol for preparation of permanent mounts[12].

### Morphological observations

Typological characters of nematodes were observed by light microscopy, including the stomatal, pharyngeal and male tail characters according to Sudhaus & Fürst von Lieven[13] for diplogastrids. Measurements and drawings of nematodes were conducted with the aid of a drawing tube attached to a microscope (Leica DM2500) and a stage micrometer. The morphometric data was processed using Excel software[14]. Photomicrographs were taken using a camera (Nikon DS-Fi1) attached to a microscope (Nikon ECLIPSE 80i) and edited using Adobe Photoshop CS6.

### Molecular profiles

Ten nematode males were separately picked into distilled water and their identity was confirmed with light microscopy before being placed into 50 μL of worm lysis buffer (WLB) containing Proteinase K for DNA extraction[15]. DNA samples were stored at –20°C until used as PCR templates.

Primers for SSU amplification were forward primer 18S965 (5’-GGCGATCAGATACCGCCCTAGTT-3’) and reverse primer 18S1573R (5’-TACAAAGGGCAGGGACGTAAT-3’)[16]. Primers for LSU amplification were forward primer D2a (5’-ACAAGTACCGTGAGGGAAAGTTG-3’) and reverse primer D3b (5’-TGCGAAGGAACCAGCTACTA-3’)[17]. The 25 μL PCR was performed using Dream *Taq* Green PCR Master Mix DNA polymerase (Thermo Fisher Scientific (China) Co. Ltd., Shanghai, China) according to the manufacturer’s protocol. The thermal cycler program for PCR was as follows: denaturation at 95°C for 5 min, followed by 35 cycles of denaturation at 94°C for 30 s, annealing at 55°C for 45 s, and extension at 72°C for 2 min. A final extension was performed at 72°C for 10 min[18]. PCR products were cleaned using ExoSap-IT (Affymetrix Inc., Santa, Clara, CA) according to the manufacturer’s protocol, and then sequenced by BGI Tech Solutions (Beijing Liuhe) Co., Ltd, Beijing, China using an ABI PRISM 3730 sequencing system.

The rDNA SSU and LSU sequences from the new species were deposited into the GenBank database under the accession numbers MG729403 and MG729404, and compared with other nematode species in GenBank using the BLAST homology search program. The most similar sequences were downloaded for phylogenetic analysis. DNA sequences were aligned by Mega5.05[19]. The model of base substitution in the SSU and LSU sets were evaluated using MODELTEST version 3.06[20]. The Akaike-supported model, the proportion of invariable sites, and the gamma distribution shape parameters and substitution rates were used in phylogenetic analyses. Bayesian analysis was performed to confirm the tree topology for each gene separately using MrBayes 3.1.0[21] running the chain for 1,000,000 generations and setting the ‘burn in’ at 1,000. The Markov Chain Monte Carlo methods within a Bayesian framework was employed to estimate the posterior probabilities of the phylogenetic trees[22] using the 50% majority-rule.

### Nomenclatural acts

The electronic vision of this paper meets the requirements of the amended international code of zoological nomenclature (ICZN), and therefore the new name contained herein is available under that code from the electronic vision of this paper. This published work and the contained nomenclatural acts have been registered in the online registration system for the ICZN in ZooBank. The ZooBank LSID (life science identifiers) for this publication is: urn:lsid:zoobank.org:pub:8402E565-4C3D-4676-87D1-C06EC261634E. The related LSID information can be viewed through any standard web browser by appending the LSID to the prefix "http://www.zoobank.org/References/".

## Results

Parasitodiplogaster religiosae* Zeng, Zeng, Zhang, Ye, Cheng, Kanzaki & Giblin-Davis n. sp.

urn:lsid:zoobank.org:act:4A50A382-CC42-4279-A651-50F7EAD16AFE

(Figs 1–4)

**Fig 1:**
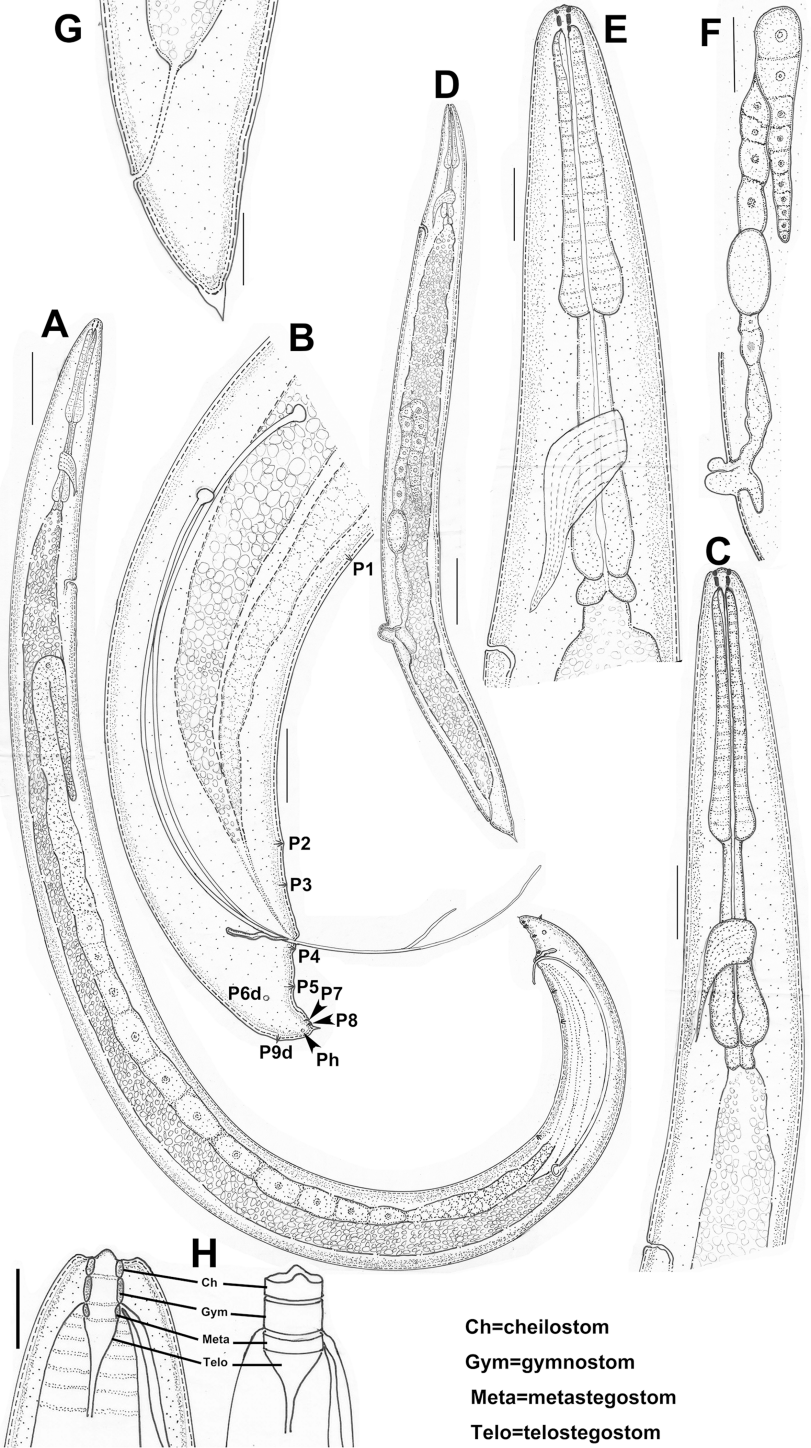
Adult male and female of *Parasitodiplogaster religiosae* n. sp. in lateral view. (A) Entire male. (B) Male tail. (C) Anterior body of male. (D) Entire female. (E) Anterior body of female. (F) Reproductive system of female. (G) Female tail. (H) Stoma and stomatal elements (lateral view) (Scale bars: A,F = 50μm; B,C,E,G = 20μm; D = 100μm; H = 10μm).

**Fig 2:**
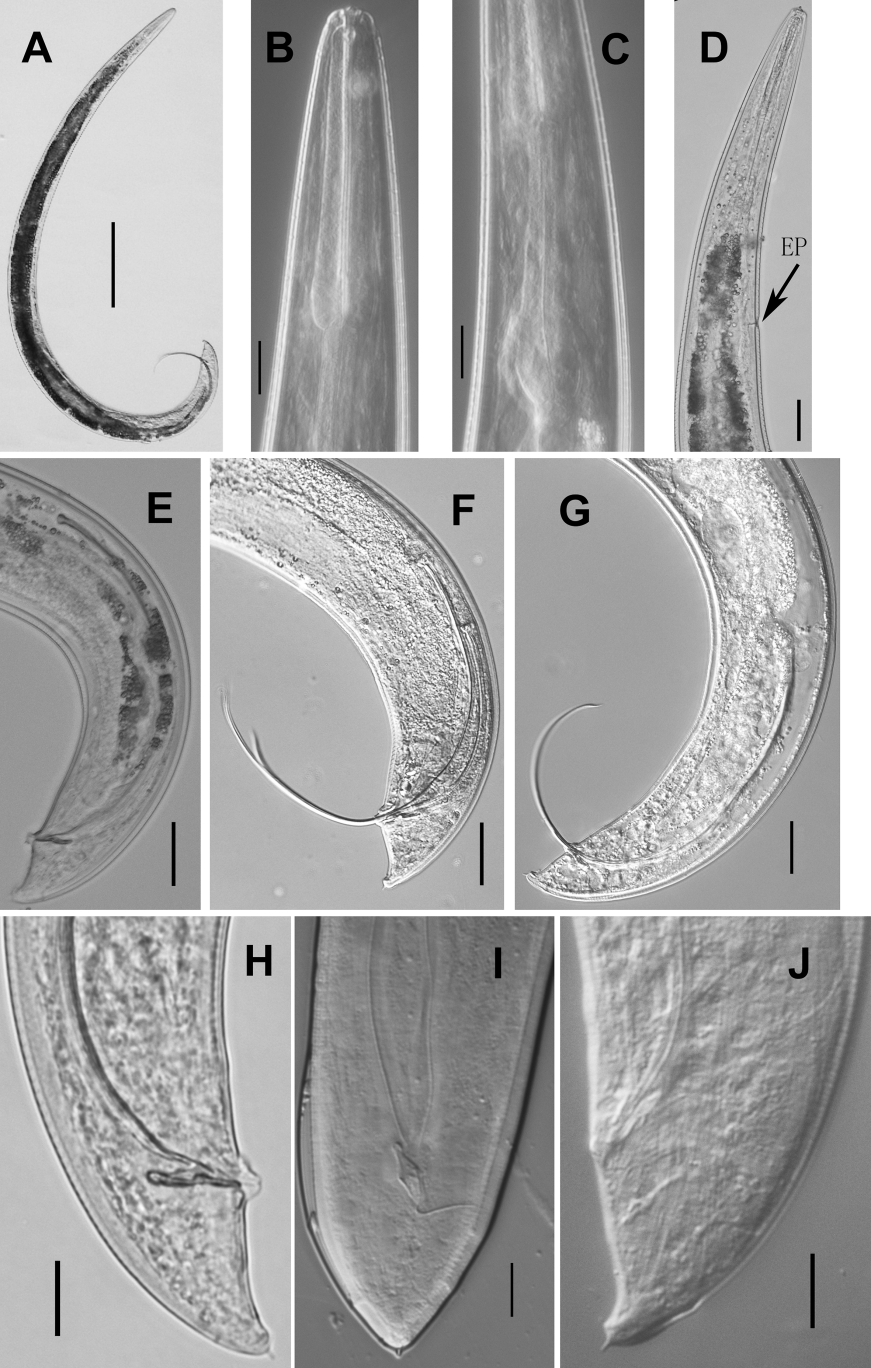
Adult male of *Parasitodiplogaster religiosae* n. sp. (A) Entire body. (B and C) Pharynx. (D) Excretory pore (EP). (E-G) Spicule. (H) Gubernaculum in lateral view. (I) Gubernaculum in ventral view. (J) Tail (Scale bars: A = 100μm; B,C,H,I,J = 10μm; D-G = 20μm).

**Fig 3:**
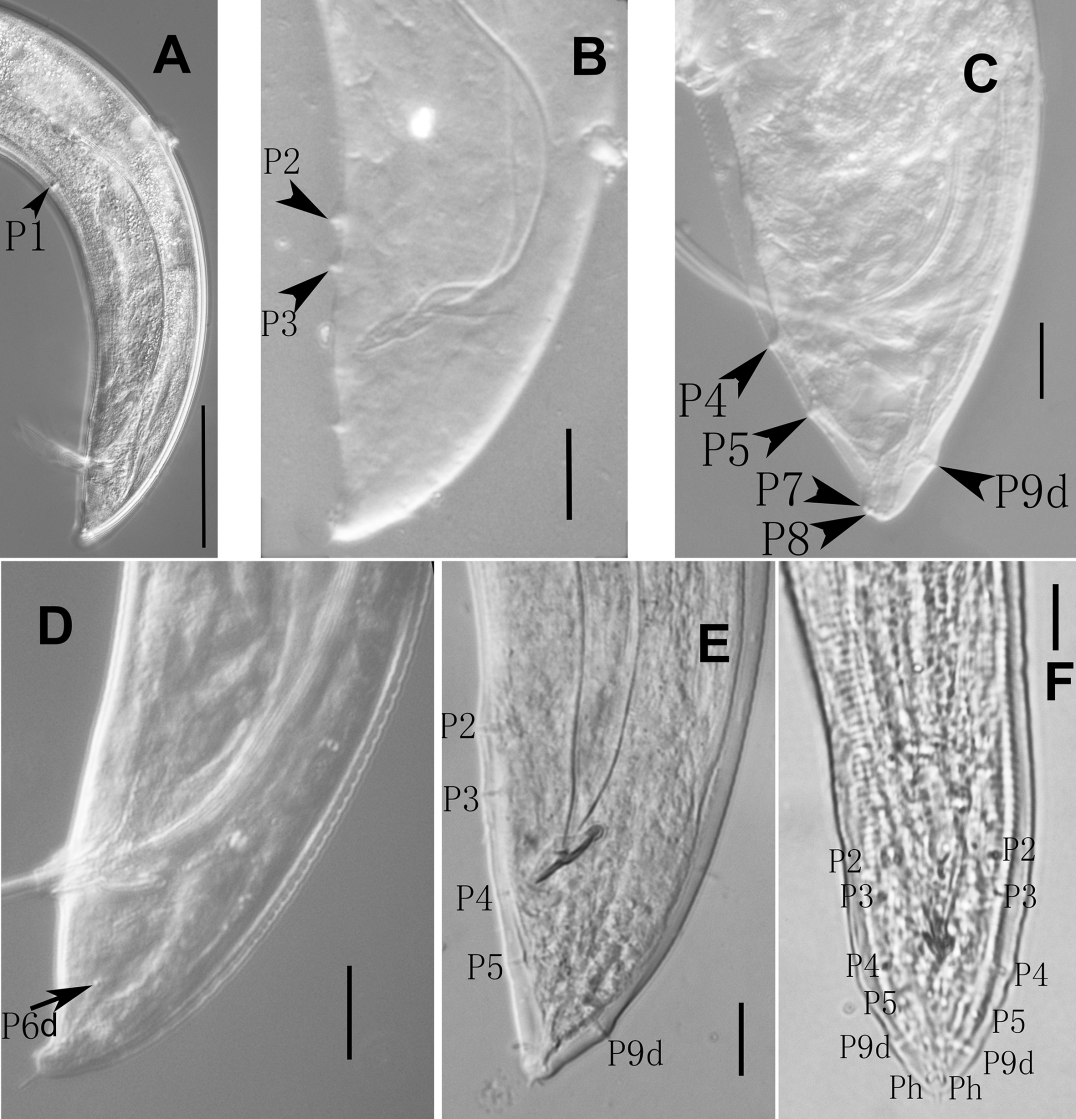
Male papillae of *Parasitodiplogaster religiosae* n. sp. (Scale bars: A = 50μm; B-F = 10μm).

**Fig 4:**
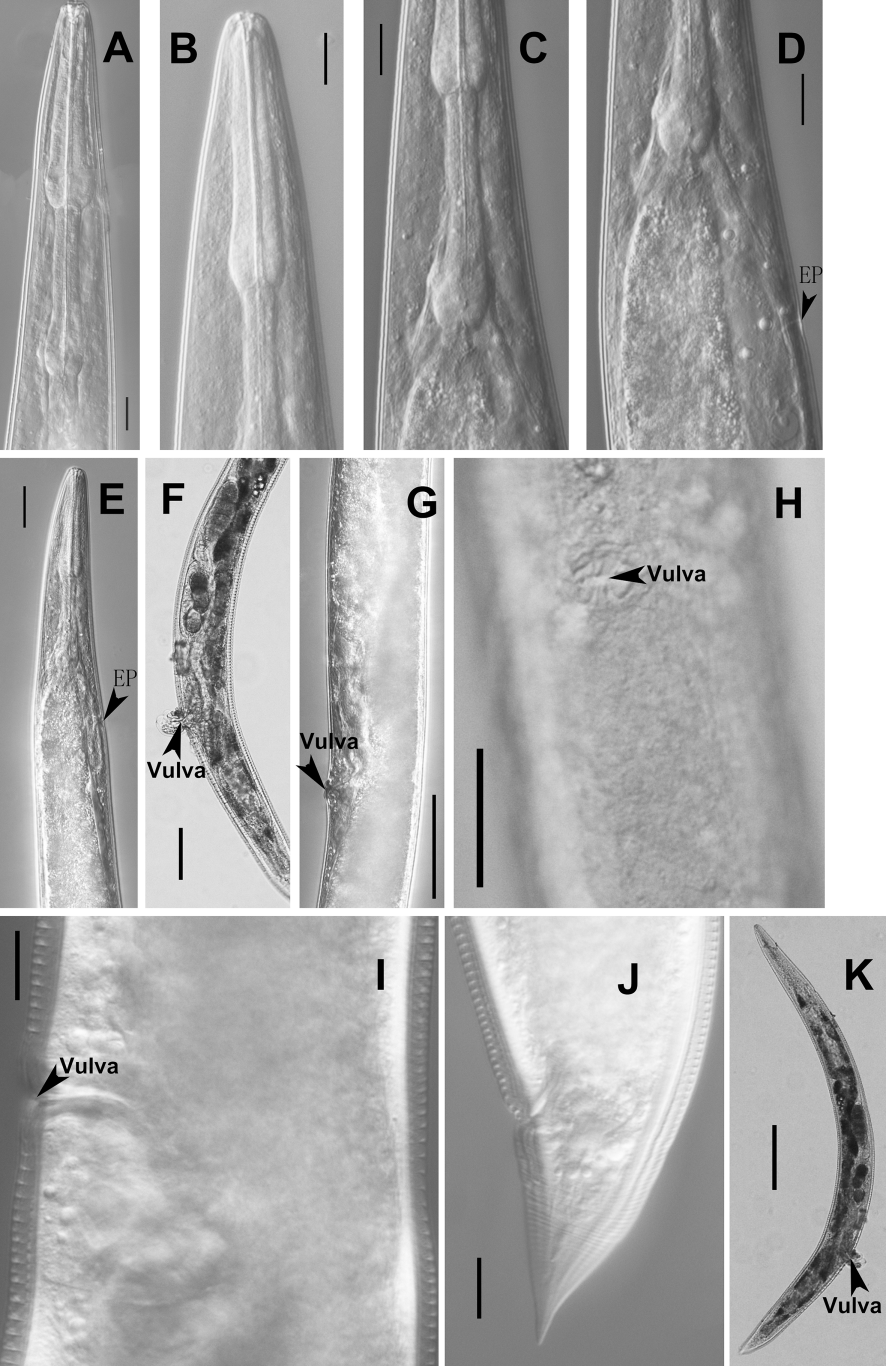
Adult female of *Parasitodiplogaster religiosae* n. sp. (A-C) Pharynx. (D and E) Excretory pore (EP). (F) Reproductive system. (G and I) Vulva in lateral view. (H) Vulva in ventral view. (J) Tail. (K) Entire body (Scale bars: A-D, H-J = 10μm; E,G = 20μm; F = 50μm).

### Description

#### Male (from figs)

Body large, 870–1210 μm long, ventrally arcuate, “L”-shaped when heat-killed(Fig 1A and Fig 2A). Cuticle finely annulated, 2 μm at mid-body. Lateral field not observed under light microscopy (LM). Head not offset(Fig 2B) with four cephalic papillae and six lip sectors, each sector bearing one small labial sensillum. Stoma consisting of a ring-like cheilostom with two indistinct triangular anteriolateral projections (hard to see under light microscopy), a tube-like gymnostom, and a funnel-like stegostom surrounded by anterior pharyngeal tissue(Fig 1H). Cheilostom short, a little wider than gymnostom and stegostom(Fig 1C), tear-shaped dots just posterior to stomatal opening in lateral view. Gymnostom short cuticular tube, twice the length of cheilostomatal element without the projections(Fig 1H). Stegostom slightly narrower than gymnostom, consisting of three sections, *i*.*e*., pro-mesostegostom, metastegostom and telostegostom(Fig 1H). Pro-mesostegostom not clearly defined under LM, metastegostom short sclerotised tube-like without teeth, denticles or ridges, telostegostom narrow and simple funnel-shaped connecting metastegostom and pharynx(Fig 1H). Anterior pharynx (procorpus + metacorpus) muscular, procorpus cylindrical, widening into slightly pyriform metacorpus(Fig 1C and Fig 2B). Posterior pharynx (isthmus + postcorpus) glandular, about equal to anterior pharynx in length(Fig 1C and Fig 2C). Cardia present(Fig 1C and Fig 2C). Hemizonid not observed. Nerve ring surrounding middle of isthmus(Fig 1C). Excretory pore opening behind base of terminal bulb, 142–180 μm from anterior end(Fig 1A and Fig 2D). Testis on left side of intestine, extended, sometimes reflexed inwardly (to right), spermatocytes multiple rows arranged in anterior part of testis, and well-developed spermatocytes arranged in single row from mid-testis, sperm amoeboid, *vas deferens* not clearly separated from male genital tract(Fig 1A). Spicules “L”-shaped or “C”-shaped when heat-killed, separate, paired, very long and thin, manubrium rounded, obviously offset from needle-like lamina(Fig 1B and Fig 2E–2G). Gubernaculum slender, anterior end bluntly rounded, and slightly ventrally bent, dorsally curved at mid-point, forming elongated “ε”-shaped, one-tenth length of spicules in lateral view, rhomboid-like in ventral view(Fig 1B and Fig 2H). Tail tapering conoid, one cloacal body diam. long, with cloacal protuberance, tail tip digital with short sharp mucron(Fig 1B and Fig 2J). Bursa absent(Fig 1A and 1B and Fig 2E–2H and 2J). Nine pairs of subventral, lateral and dorsal genital papillae present(Fig 1B). P1, P2 and P3 pairs at 3.8, 0.8 and 0.4 cloacal body diam. anterior to cloacal slit, respectively(Fig 1B and Fig 3A and 3B), P4 pair just posterior to cloacal slit, at level of gubernaculum(Fig 1B and Fig 3C–3E and 3F), P5 pair at 0.4 cloacal body diam. posterior to cloacal slit(Fig 1B and Fig 3C–3E and 3F), P6d pair on lateral side, 0.5 cloacal body diam. posterior to cloacal slit(Fig 1B and Fig 3D), and P7 and P8 almost contiguous at level of phasmid near tail tip(Fig 1B and Fig 3C). P9d on dorsal side, 12 μm from tail end (Fig 1B and Fig 3C–3E and 3F). Phasmids pore-like(Fig 1B). Measurements are listed in Table 1.

**Table 1:** *Parasitodiplogaster religiosae* n. sp. morphometrics of males and females mounted in 4% formalin. All measurements in μm and in the format: mean ± S.D. (Range).

Character	Male (Holotype)	Males (Paratypes)	Females (Paratypes)
	(n = 1)	(n = 10)	(n = 10)
L	1050.0	1030.0 ±125.2(870.0–1210.0)	1220.5 ± 226.5(998.0–1605.0)
a	21.0	20.0 ± 1.5(18.5–22.4)	18.8 ± 2.4 (14.3–21.5)
b	8.1	7.5 ± 0.7(6.5–8.4)	8.2 ± 1.1 (6.8–9.8)
c	30.0	30.9 ± 3.0(27.2–35.3)	19.5 ± 3.0 (14.3–22.9)
c’	1.2	1.1 ± 0.0(1.1–1.2)	2.0 ± 0.1(1.8–2.2)
V	-	-	65.5 ± 2.2 (61.8–68.6)
Body diam. (GBD)	50.0	51.5 ± 4.8(47.0–60.0)	66.2 ± 16.2(53.0–92.0)
Pharynx length	130.0	136.8 ± 11.4(124.0–156.0)	148.2 ± 9.3 (140.0–164.0)
Spicule length	176.0	181.4 ± 31.1(142.0–228.0)	-
Gubernaculum length	15	16.0 ± 2.2(14.0–19.0)	-
Anal body diam. (ABD)	30.0	30.2± 1.0(29.0–32.0)	31.7 ± 6.6 (27.0–44.0)
Tail length	35.0	33.3 ± 1.8(32.0–36.0)	63.8 ± 15.4 (52.0–92.0)
Excretory pore from anterior end	142.0	151.2 ± 14.4(142.0–180.0)	177.2 ± 22.1 (153.0–206.0)
Anterior pharynx (A)	70.0	73.7 ± 5.8(66.0–83.0)	81.3 ± 3.8 (76.0–87.0)
Posterior pharynx (P)	60.0	63.2 ± 5.9 (58.0–73.0)	66.8 ± 6.4 (60.0–77.0)
P/A ratio	0.9	0.9 ± 0.0(0.8–0.9)	0.8 ± 0.1 (0.7–0.9)
Gubernaculum length*100/ spicule length	8.5	9.0 ± 0.7(8.3–9.9)	-

#### Female (from figs)

Body curved ventrally or dorsally when heat-killed(Fig 1D and Fig 4K). Cuticle, pharynx and stoma morphology similar to male(Fig 1E and Fig 4A–4E). Vulva posteriorly located(Fig 1D and Fig 4K). Anterior reproductive tract situated on the right side of intestine, prodelphic, well-developed with eggs and reflexed(Fig 4F). Oocytes arranged in a single file. Posterior reproductive tract manifests as short post uterine sac (PUS), 45–50 μm long(Fig 1D and Fig 4F and 4G). Vulva protuberant, four large vaginal gland cells present, 40–45 μm long each(Fig 4F, 4G and 4K). Vagina not perpendicular to body surface(Fig 4G). Rectum about 0.5–1.0 anal body diam. long(Fig 4J). Tail tapering, conoid with a sharp tip, no mucron present(Fig 1G and Fig 4J). Phasmid pore-like, near tail tip. Measurements are listed in Table 1.

### Type host and locality

*Parasitodiplogaster religiosae* n. sp. was collected from phase B-C syconia from a *Ficus religiosa* tree at the Guangxiao Temple (23°07’55.81”N, 113°15’3.47”), Guangzhou, China, on May 26, 2016 by the first author.

### Type materials

Holotype male, one male paratype and one female paratype deposited in the Department of Nematology, University of California, Riverside, CA, USA. One male paratype and one female paratype deposited in the USDA Nematode Collection, Beltsville, MD, USA. The remaining paratypes deposited at the Plant Pathology Laboratory, Department of Plant Protection, Zhongkai University of Agriculture and Engineering, Guangzhou, P. R. China.

### Diagnosis and relationships

**Fig 5:**
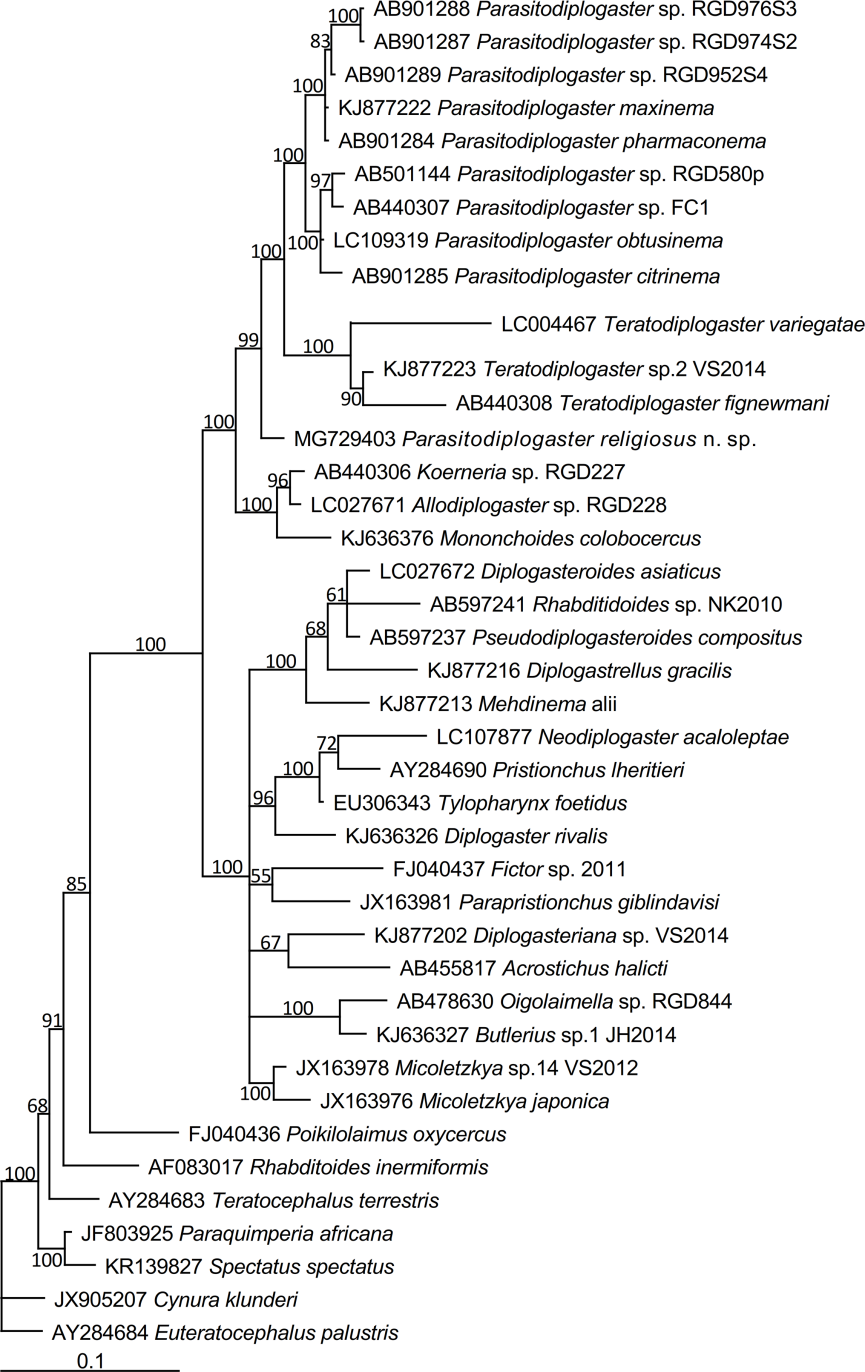
Bayesian tree inferred from SSU rDNA sequences. Posterior probability values exceeding 50% are given on appropriate clades. GenBank and strain ID associated with DNA sequences are shown on the left and right of the species name respectively.

**Fig 6:**
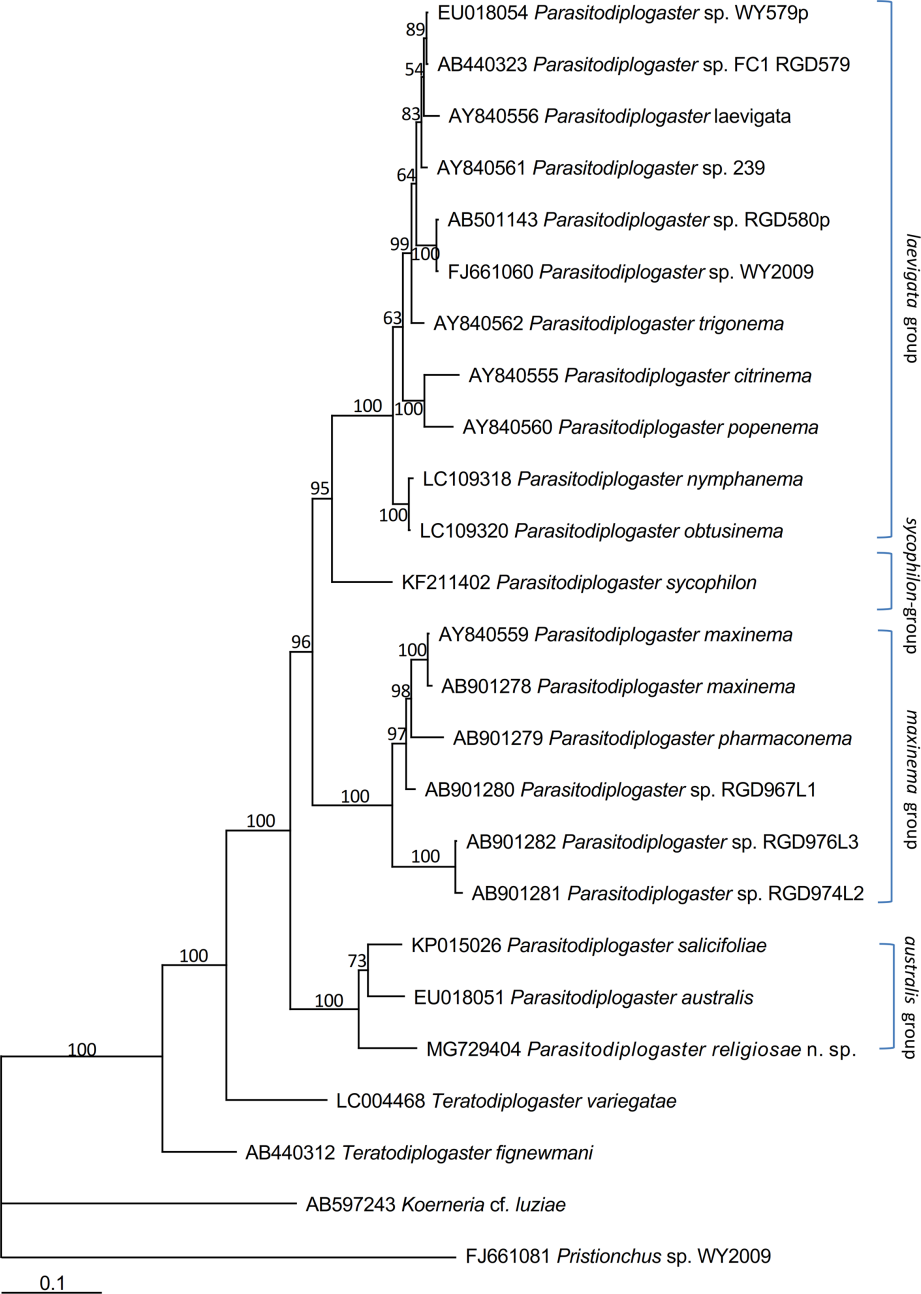
Bayesian tree inferred from LSU D2-D3 rDNA sequences. Posterior probability values exceeding 50% are given on appropriate clades. GenBank and strain ID associated with DNA sequences are shown on the left and right of the species name respectively.

*Parasitodiplogaster religiosae* n. sp. is characterised by possessing the longest and thinnest spicule of all currently described males in the genus, an elongated laterally “ε-shaped” and ventrally rhomboid-like gubernaculum, a stoma without teeth, consisting of a ring-like cheilostom with indistinct squared anteriolateral projections, a tube-like gymnostom and a funnel-like stegostom, monodelphic and a mean vulval position of 65.5%. There are three pre-cloacal and six post-cloacal male genital papillae with the arrangement P1, P2, P3, (C, P4), P5, P6d, P7, P8, P9d, Ph. Its status as a distinct species is corroborated by DNA sequences of SSU and D2-D3 LSU (Figs 5 and 6).

The genus *Parasitodiplogaster* is separated into several species groups that align with different *Ficus* subsections and their associated pollinators[9]. The new species belongs to the *australis-*group associated with the *Ficus* subsection Urostigma and the fig wasp genus *Platyscapa*. Members of this group typologically share a relatively simple stoma (with no metastegostomatal teeth), single female gonad and very short and conical tail of males[9]. Currently, the group contains two species, *P*. *australis* and *P*. *salicifoliae*. The molecular phylogenetic status of the species also supports its inclusion in the group (Figs 5 and 6).

*Parasitodiplogaster australis* sp. n. is differentiated from all other species of the genus by having females with only one gonad, males with C-shaped spicules with an arcuate, slender gubernaculum, characteristic arrangement of the male caudal papillae and DNA sequence data. The generic diagnosis of *Parasitodiplogaster* is emended to include loss of a female gonad.

Based upon the possession in females of a monodelphic ovary, *P*. *religiosae* n. sp. is closest to *P*. *salicifoliae* and *P*. *australis*. However, *P*. *religiosae* n. sp. differs from *P*. *salicifoliae* by possessing a different shape spicule (needle-like *vs* sickle-like), a longer spicule (142–228 *vs* 36–54 μm), a shorter gubernaculum (14–19 *vs* 19–32 μm), a shorter pharynx (124–156 *vs* 164–247 μm), a shorter tail (32–36 *vs* 44–63 μm), a higher A/P ratio (0.8–0.9 *vs* 0.45–0.72) and a different gubernaculum shape (elongated “ε”-shaped *vs* “C”-shaped), different EP position (posterioriad to pharyngointestinal junction *vs* at level of the pharyngointestinal junction) and number and arrangement of caudal papillae (9 *vs* 8 pairs, <P1, P2, P3, (C, P4), P5, P6d, P7, P8, P9d, Ph> *vs* <P1, P2, P3, (C, P4), P5, P6, P7, P8, P9d, Ph>) in males, different tail tip (no mucron *vs* with small mucron) in females. It differs from *P*. *australis* by having a longer spicule (142–228 *vs* 40–49μm), a shorter gubernaculum (14–19 *vs* 20–30 μm), a higher c ratio (1.1–1.2 *vs* 0.63–1.12) and A/P ratio (0.8–0.9 *vs* 0.5–0.8), and a different gubernaculum shape (elongated “ε”-shaped *vs* arcuate), and number and arrangement of caudal papillae (9 *vs* 7 pairs, <P1, P2, P3, (C, P4), P5, P6d, P7, P8, P9d, Ph> *vs* <P1, P2, (C, P3), P4, P5d, P6, P7, Ph>) in males. *Parasitodiplogaster religiosae* n. sp. is also distinguished from *P*. *australis* and *P*. *salicifoliae* based upon the molecular sequence differences in LSU D2-D3 (10.4%, 8.7%, respectively).

### Molecular phylogenetic relationships

Partial SSU and D2-D3 LSU expansion domains were sequenced for molecular characterization. Bayesian analysis of the relative placement of *Parasitodiplogaster religiosae* n. sp. among other *Parasitodiplogaster* species was performed. The tree inferred from SSU (Fig 5) using *Cynura klunderi* Murphy, 1965 and *Euteratocephalus palustris* (de Man, 1880) Andrássy, 1958, as outgroups suggested that: *i*) all the selected species (taxon) are in a monophyletic clade with 100% posterior probability (pp); *ii*) *Parasitodiplogaster religiosae* n. sp., nine populations of *Parasitodiplogaster* and three populations of *Teratodiplogaster* and othr three populations (*Allodiplogaster josephi* Kanzaki, Ragsdale & Giblin-Davis, 2015[23], *A*. *seani* Kanzaki, Ragsdale & Giblin-Davis, 2015[23] and *A colobocerca* (Andrássy, 1964[24]) Kanzaki, Ragsdale & Giblin-Davis, 2014[25]) are grouped in a monophyletic clade with 100% pp; *iii*) *Parasitodiplogaster religiosae* n. sp. is clustered in a highly supported (99% pp) monophyletic clade with the above-mentioned nine *Parasitodiplogaster* populations and three *Teratodiplogaster* populations. Unfortunately, the other members of the *australis*-group (*P*. *australis* and *P*. *salicifoliae*) have not been sequenced for SSU and were not available for analysis.

The tree inferred from D2-D3 of LSU (Fig 6) using *Koerneria* cf. *luziae* and *Mononchoides* sp. WY2009 (= *Pristionchus* sp.; see Susoy et al.[26]) as outgroups suggested that: *i*) *Parasitodiplogaster religiosae* n. sp., all 20 of the selected species (taxa) of *Parasitodiplogaster* and two species of *Teratodiplogaster* are in a monophyletic clade with 100% pp; *ii*) all the selected populations of *Parasitodiplogaster* are separated into four groups including the *laevigata*-group associated with figs and fig wasps associated with the subsection Americana, the *maxinema*-group (subsection Pharmacosycea), the *australis*-group (subsection Urostigma) and the *sycophilon*-group (subsection Galoglychia), and *P*. *religiosae* n. sp. is clustered in the *australis*-group together with *P*. *australis* and *P*. *salicifoliae* with 100% pp.

## Discussion

Morphologically *P*. *religiosae* n. sp. belongs to the *Parasitodiplogaster australis-* group (*P*. *australis* + *P*. *salicifoliae*) which have only one ovary in the females (*vs* two ovaries in all of the other described species in the genus). However, it possesses the longest spicule relative to all other described species in the genus (by more than 2X). Phylogenetically, *P*. *religiosae* n. sp., *P*. *australis* and *P*. *salicifoliae* are putative sisters in a single clade and clustered into the “*australis*-group” (Fig 6), but its sequences are unique. Even though the “*australis*-group” comes out of figs from the same Section and Subsection (Urostigma; Urostigma) their collection sites are geographically widely disparate (from China, Australia, and South Africa, respectively). The 10.4% of molecular sequence differences in D2-D3 LSU between *P*. *religiosae* n. sp. and *P*. *australis*, and 8.7% between and *P*. *salicifoliae* helped to confirm that *P*. *religiosae* n. sp. is a distinct species from *P*. *australis* and *P*. *salicifoliae*. The genus *Parasitodiplogaster* was phylogenetically separated into four groups (Fig 6) as previously reported[9–11]. *Parasitodiplogaster religiosae* n. sp. is clearly clustered into the “*australis*-group” with *P*. *australis* and *P*. *salicifoliae* (Fig 6). The species within this group share the same *Ficus* subsection Urostigma and fig wasp genus *Platyscapa* and the characters of possessing stomas without teeth and females being monodelphic when compared with *Parasitodiplogaster* species in the other three groups. The possession or lack of metastegostomatal of teeth is confusing because Poinar[2] and Poinar & Herre[4] reported no teeth in the other three groupings, but re-descriptions have confirmed this to be incorrect[8,10,11,27]. In addition, Bartholomaeus et al.[6] reported two large metastegostomatal teeth in *P*. *australis* which upon subsequent re-examination of fresher specimens showed the putative teeth to actually be cheilostomal anteriolateral projections, not metastegostomatal teeth (Nutsumi Kanzaki and Robin M. Giblin-Davis Unpubl. Obs.). Among species of the *australis-*group there are distinct differences in the spicule, gubernaculum and stomatal morphology and genital papillae number, *e*.*g*., the longest and thinnest spicule in *P*. *religiosae* n. sp., and 9 pairs of the papillae in *P*. *religiosae* n. sp. *vs* 8 in *P*. *salicifoliae* and 7 in *P*. *australis*. Based on the morphological, molecular and host association data, species in the *australis-*group continue to appear to comprise a naturally derived lineage within Parasitodiplogaster with generic attributes. Wöhr et al.[9] suggested that *P*. *salicifoliae* and *P*. *australis* might comprise a new genus. The inferred placement of *P*. *religiosae* n. sp. further supports this hypothesis, but more extensive biogeographical study, fig (subsection Urostigma) and fig wasp (*Platyscapa*) host sampling, and SSU sequencing of *P*. *salicifoliae* and *P*. *australis* are required for this action in the future.
